# Canadian Society of Nephrology Commentary on the 2025 Kidney Disease Improving Global Outcomes Clinical Practice Guidelines for Autosomal Dominant Polycystic Kidney Disease

**DOI:** 10.1177/20543581261455635

**Published:** 2026-06-03

**Authors:** Matthew B. Lanktree, Nick Ashawasega, Micheli Bevilacqua, Daniel G Bichet, Guillaume Bollée, Pierre-Antoine Brown, Louis Girard, Paul Goodyer, Jay Hingwala, Mathieu Lemaire, Philip McFarlane, Louise Moist, York Pei, Normand Proulx, Clara Schott, Steven Soroka, Caitlyn Vlasschaert, Nicholas Watkins, Ahsan Alam

**Affiliations:** 1 Division of Nephrology, St. Joseph’s Healthcare Hamilton, Hamilton, Ontario & Divisions of Medicine and Health Research Methods, Evidence, and Impact, 3710 McMaster University, Hamilton, ON, Canada; 2 Patient Partner; 3 Division of Nephrology, Department of Medicine, 8166 University of British Columbia, Vancouver, BC, Canada; 4 Hôpital Du Sacré-Coeur de Montréal, 5622 University of Montreal, Montreal, QC, Canada; 5 Centre Hospitalier de L’Université de Montréal, Institut de Recherche Clinique de Montréal, 5622 University of Montreal, Montreal, QC, Canada; 6 Division of Nephrology, Department of Medicine, 6363 University of Ottawa & the Ottawa Hospital Research Institute, Ottawa, ON, Canada; 7 Division of Nephrology, Department of Medicine, Cumming School of Medicine, 70401 University of Calgary, Calgary, AB, Canada; 8 Department of Pediatrics, 5620 McGill University, Montreal, QC, Canada; 9 Division of Nephrology, Winnipeg Health Sciences Centre, 8664 University of Manitoba, Winnipeg, MB, Canada; 10 Nephrology Division, The Hospital for Sick Children, Toronto, ON, Canada; 11 Nephrology Division, St Michael’s Hospital, Toronto, ON, Canada; 12 Division of Nephrology, Schulich School of Medicine and Dentistry, 6221 Western University, London, ON, Canada; 13 Division of Nephrology, 7989 University Health Network, Toronto, ON, Canada; 14 Division of Nephrology, 7938 University of Toronto, Toronto, ON, Canada; 15 Division of Nephrology, Department of Medicine, Centre Intégré de Santé et de Services Sociaux de L’Outaouais, Gatineau, QC, Canada; 16 Department of Biochemistry, Schlich School of Medicine and Dentistry, 6221 University of Western Ontario, London, ON, Canada; 17 Halifax Division of Nephrology, Department of Medicine, Dalhousie University, Halifax, NS, Canada; 18 Division of Nephrology, Department of Medicine, 4257 Queen’s University, Kingston, ON, Canada; 19 Department of Molecular Genetics, 7938 University of Toronto, Toronto, ON, Canada; 20 Division of Nephrology, 54473 McGill University Health Centre, Montreal, QC, Canada

**Keywords:** ADPKD (autosomal dominant polycystic kidney disease), clinical practice guidelines, genetics

## Abstract

**Purpose of review:**

1) Provide a Canadian perspective on the 2025 Kidney Disease Improving Global Outcomes (KDIGO) Autosomal Dominant Polycystic Kidney Disease (ADPKD) guidelines; 2) identify challenges and nuances in applying these guidelines in Canada; 3) highlight shifts in expert practice points for Canadian care providers; 4) outline opportunities for research, knowledge translation, and quality improvement in Canada.

**Sources of information:**

The KDIGO 2025 Clinical Practice Guideline Update for the management of ADPKD, as well as a survey and discussion by Canadian experts in ADPKD.

**Methods:**

The co-chairs invited stakeholders from the Canadian ADPKD community to ensure national representation, including adult and pediatric clinicians, trainees, a genetic counselor, and a patient partner with an Indigenous perspective. Members were surveyed to identify key practice points. Subgroups reviewed issues and drafted discussion topics. All members reviewed the final draft.

**Key Findings:**

The committee commented on recommendations with nuance for Canadian practitioners, especially on multidisciplinary care, challenges with genetic testing, and the use of CKD therapies like sodium-glucose transport protein 2 (SGLT2) inhibitors in ADPKD.

**Limitations:**

The committee relied on the evidence summaries produced by KDIGO and the experience and knowledge of committee members. The committee did not replicate or update the systematic reviews.

## Abstract (fr)

**Objectifs de la revue:**

1) Présenter une perspective canadienne sur les lignes directrices KDIGO (Kidney Disease Improving Global Outcomes) 2025 relatives à la polykystose rénale autosomique dominante (ADPKD); 2) cerner les défis de leur application au Canada; 3) souligner les changements dans les pratiques expertes pour les prestataires de soins canadiens; et 4) dégager des pistes de recherche, de transfert des connaissances et d’amélioration de la qualité au Canada.

**Sources de l’information:**

La mise à jour des lignes directrices de pratique clinique KDIGO 2025 pour la prise en charge de l’ADPKD, ainsi qu’un sondage et une discussion menés par des spécialistes canadiens de l’ADPKD.

**Méthodologie:**

Les coprésidents du comité ont invité des intervenants de la communauté canadienne de l’ADPKD, ce qui assure une représentation nationale, notamment des cliniciens en néphrologie adulte et pédiatrique, des stagiaires, un conseiller en génétique et un patient partenaire apportant une perspective autochtone. Les membres ont été interrogés afin de dégager les principaux points de pratique. Des sous-groupes ont examiné les enjeux et préparé les sujets de discussion. Tous les membres ont révisé la version finale.

**Principaux résultats:**

Le comité a nuancé certaines recommandations pour le contexte canadien, notamment en ce qui concerne les soins multidisciplinaires, les difficultés associées aux tests génétiques et l’utilisation de traitements de l’IRC, comme les inhibiteurs du cotransporteur sodium-glucose de type 2 (iSGLT2) pour l’ADPKD.

**Limites:**

Le comité s’est appuyé sur les synthèses de données probantes de KDIGO ainsi que sur l’expérience et les connaissances des membres du comité. Le comité n’a pas reproduit ou mis à jour les revues systématiques.

## Introduction

Kidney Disease Improving Global Outcomes (KDIGO) aims to improve the care and outcomes of kidney disease patients worldwide through promotion, coordination, collaboration, and integration of initiatives to develop and implement clinical practice guidelines. The 2025 KDIGO Clinical Practice Guidelines for the evaluation, management, and treatment of Autosomal Dominant Polycystic Kidney Disease (ADPKD) were an enormous effort of 24 ADPKD and evidence review experts from around the globe to form a foundation of expectations and suggestions for the standardized care of patients with ADPKD.^1^ The document included 22 graded recommendations arising out of a systematic review of evidence, and 248 “practice points” representing consensus-based statements representing the expert judgment of the KDIGO workgroup. The KDIGO ADPKD guidelines include more than 240 pages of supporting information, providing an invaluable resource for experts and trainees alike.

Arising from the KDIGO ADPKD guidelines was the recognition of specific intricacies in applying recommendations and practice points within the Canadian context. For example, access to technology, expertise, infrastructure, and resources may be different in Canada than in other international settings, and even variation within Canada itself exists. Cultural and ancestral differences in the Canadian context require specific attention. Opportunities for future research, knowledge translation, and quality assurance implementation exist in Canada. We sought to engage Canadian opinion leaders and stakeholders based on their knowledge of the Canadian ADPKD community, aiming for national representation, including academic and community practitioners, adult and pediatric nephrologists, as well as medical trainees, a genetic counsellor, and a patient partner, to evaluate the 2025 KDIGO ADPKD clinical practice guidelines from the Canadian perspective. The scope of the current commentary is not to reevaluate all recommendations and practice points, but to raise and comment on issues of particular interest to committee members. The intended audience is nephrologists and those with an interest in issues of importance for the care of those with ADPKD in Canada.

### Review Process for Canadian ADPKD Commentary

The Canadian Society of Nephrology (CSN) Clinical Practice Guideline Committee previously developed standards for adapting existing guidelines.^2^ A committee was established, consisting of two Co-Chairs appointed by the CSN (Drs. Lanktree & Alam), and a multidisciplinary group formed with *ad hoc* invitations to a geographically diverse group of CSN members with expertise and clinical experience in ADPKD. Members include adult and pediatric nephrologists with clinical and content expertise, including methodological guideline expertise. Additional relevant physician specialty groups, patients and family members, and members of the allied health community (pharmacists, nurses, dieticians, genetic counsellors) were invited to participate or consulted.

**Figure 1.**
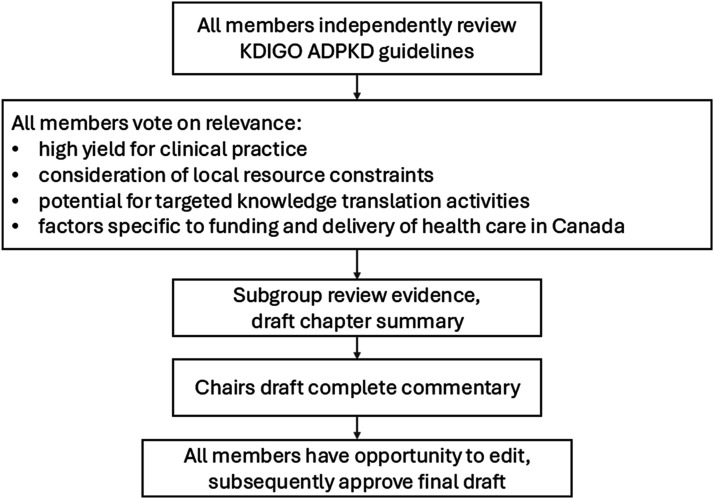
Overview of process for Canadian commentary on KDIGO ADPKD guidelines

The working group systematically reviewed all recommendations and practice points and identified those requiring further exposition and inclusion in the Commentary. Examples of recommendations or practice points that are relevant for interpretation and application may include those with: i) high yield for clinical practice, ii) consideration of resource constraints, iii) potential for targeted knowledge translation activities relevant to the local, regional, and national context, and iv) consideration of factors specific to funding and delivery of health care in Canada (Figure 1).

Before the first working group meeting, all members completed a survey to determine the need for discussion of each recommendation and practice point. Responses from the survey, including an opportunity for free-text comment, were collated and disseminated in advance of the first working group meeting. Practice points were ranked from greatest to least relevant for requiring discussion. Working subgroups were formed to evaluate each chapter, followed by a presentation and an opportunity for open discussion from the complete group. Each working group member participated in multiple subgroups based on their area of interest and focused expertise. The chairs of each working group drafted summary recommendations, followed by drafting of these discussions into the final commentary by the co-chairs. Finally, all Canadian Workgroup members were provided the opportunity to edit and approve the final manuscript.

### Current State of Care for Patients With ADPKD in Canada

**Figure 2.**
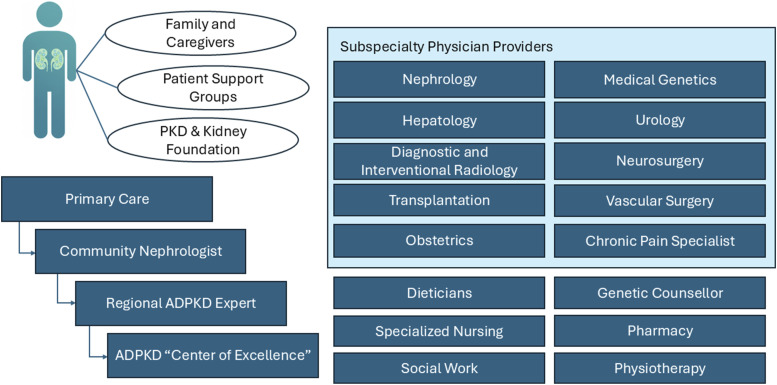
Team members in the care of patients with ADPKD

Primary care physicians often screen and manage the general care of patients with ADPKD but may lack disease-specific expertise. Driven by demand to improve targeted treatment of ADPKD, a network of specialized nephrology clinics with experience treating ADPKD has been established across Canada. These clinics typically care for patients early in the disease process with estimated glomerular filtration rate (eGFR) in the normal range. Care of renal and extra-renal manifestations of ADPKD has largely been driven by nephrologists. Development of multidisciplinary networks with an interest in ADPKD, including but not limited to radiology, medical genetics, hepatology, urology, vascular and neurosurgery, obstetrical medicine, and pain specialists, could improve care of patients with ADPKD (Figure 2).

### Chapter 1: Nomenclature, Diagnosis, Prognosis, and Prevalence

#### Terminology

The term ADPKD is the preferred term and the most used in the literature, particularly in classical cases with a defined genetic cause, modified with the involved gene name in italics when known (Practice point 1.1.4). Our workgroup highlighted the importance of stakeholder education regarding the significance of this nomenclature approach. Although it better classifies the genetic subtypes of ADPKD, it should not be equated to risk stratification or influence insurance coverage or denial of potential targeted therapies on its own. The inheritance pattern may be difficult to ascertain, particularly with missing information in the parental generation, and genetic testing can be helpful to clarify the diagnosis in uncertain cases. We feel the term polycystic kidney disease (PKD or PCKD) is appropriate in cases with multiple kidney cysts without a concrete diagnosis. As reviewed in the KDIGO document, multiple kidney cysts can be observed in many genetic and acquired conditions (Practice point 1.3.14) with differing prognosis and therapeutic implications.

**Table 1.** Resources for Self-Management in Patients With ADPKD

Resource	Description	Website
Polycystic Kidney Disease Foundation of Canada	A registered charity providing support, awareness, advocacy, research, and education	https://www.endpkd.ca/
British Columbia Renal Agency	Includes resources for all kidney diseases, but also ADPKD-specific content	https://www.bcrenal.ca/health-info/kidney-care/polycystic-kidney-disease
Mayo clinic polycystic kidney disease resource center	American-based PKD center of excellence with patient-oriented education materials	https://mcpress.mayoclinic.org/polycystic-kidney-disease/
Polycystic Kidney Disease Foundation	American-based charity with extensive resources	https://pkdcure.org/
Canada Food Guide	Updated dietary recommendations for all, but applicable to those with ADPKD	https://food-guide.canada.ca/en/

Guidelines from the American College of Medical Genetics and Genomics (ACMG) have recommended replacement of “mutation” with the term “variant” with appropriate modifiers: “pathogenic”, “likely pathogenic”, “uncertain significance”, “likely benign”, or “benign”.^3^ This tiered system has been applied in all genetic diseases, conveys confidence in the variant interpretation, and attempts to reduce genetic discrimination. Whether the variant is a truncating or non-truncating variant can be noted^4^ but is not included in the KDIGO recommended nomenclature as is not routinely used in ACMG classification (Practice point 1.1.6).

Despite efforts to define the terms, recognizing “typical”, “atypical”, or “equivocal” features for ADPKD can be challenging, particularly for inexperienced clinicians.^5-7^ High-resolution imaging using either magnetic resonance (MR) or computed tomography (CT) and genetic testing are helpful for the diagnosis and risk stratification of patients with ADPKD, particularly those with any atypical features. However, we acknowledge that access to MR and genetic testing can be limited in some parts of Canada and represents a challenge. Genetic testing may not be required for the management of cases with a clear diagnosis. However, the availability and recognition of the value for genetic testing are increasing, particularly to improve diagnostic certainty in atypical cases with eGFR decline, or in the absence of a positive family history of ADPKD, or need for diagnosis at a young age.^8,9^ Consultation or referral to a center of excellence to assist with diagnosis and risk stratification, either in person or virtually, is reasonable and frequently available across Canada.

#### Screening

Our workgroup feels that emphasis on appropriate counselling regarding the value and ramifications of ADPKD screening in asymptomatic individuals is important in Canada (Practice points 1.3.1-3). We agree that screening of adults at risk of ADPKD should first be done with ultrasound (Recommendation 1.3.1). Counselling should be provided before ultrasound-based screening, as the discovery of kidney cysts may have ramifications on insurability (life, disability, critical illness, etc.).

#### Genetic Testing

Despite initial concern of homology with *PKD1* pseudogenes, our Canadian workgroup agreed that a next-generation sequencing panel including at least *PKD1* and *PKD2* is an appropriate first-line genetic test (Practice point 1.3.15), which is available in Canada. In the context of negative genetic testing but a high clinical suspicion, referral to a tertiary center for additional testing is suggested, as genetic testing cannot exclude an inherited form of ADPKD (Practice point 1.3.18), and there is a growing list of additional genes and genetic mechanisms behind polycystic kidneys.

Our workgroup recommends that clearer pathways for access to genetic testing, genetic counselling, and Nephrology Genetics consultation are still generally required in Canada. All clinicians need to be familiar with about the benefits and harms of genetic testing in ADPKD (Practice Point 10.4). Genetic testing is particularly helpful in people an equivocal diagnosis based on kidney imaging and in those with a negative or unknown family history (Practice Point 1.3.10), living related donor for transplantation (Practice Point 1.3.11), or families with marked phenotypic variability, including pediatric onset-ADPKD (Practice Point 1.3.12).

A special feature in Canada is the Genetic Non-Discrimination Act (GNDA), which is intended to protect people from discrimination on the basis of genetic test results, particularly in non-medical areas such as insurance and employment.^10^ Genetic test results are authorized for use by medical professionals and in the context of research, with the patient’s informed consent. However, GNDA does not protect individuals from the ramifications of a positive family history of disease or the presence of early disease manifestations such as kidney cysts.

### Risk stratification

A KDIGO recommendation for Mayo imaging classification (MIC) in patients with ADPKD is commonly implemented in Canadian practice (Recommendation 1.4.2.1). We feel it is important to note that CT without contrast is acceptable for determining MIC.^11^ Our workgroup feels that recognizing kidney size is still important, but it is appropriate not to apply MIC to patients with non-*PKD1* non-*PKD2* ADPKD, as such patients represented a small minority of cases in the development of MIC (Practice point 1.4.2.5). Moreover, cases with non-*PKD1* non-*PKD2* ADPKD typically have modest kidney volumes. However, providers should be aware of the possibility of missed *PKD1* or *PKD2* variants in patients with large kidney volume ADPKD and a diagnosis of a non-*PKD1* non-*PKD2* ADPKD or ADPKD with no pathogenic variant identified.

### Prevalence

According to Canadian Institute for Health Information data, ADPKD represents 7% of incident cases of kidney failure, representing approximately 2,000 cases annually and making it the most common cause of kidney failure due to a single genetic change.^12^ ADPKD is a genetic condition and is thus present from birth, but may be asymptomatic and unrecognized well into adulthood. ADPKD is present in people of all ancestries and both sexes. Given a population prevalence of about 1 in 1,000, we can estimate approximately 40,000 Canadians are living with ADPKD.^13^ However, detailed measurement of ADPKD prevalence, particularly in underrepresented and underserved demographic groups or identify geographic areas of greater ADPKD concentration in Canada, is an area requiring further research. Hopefully, further efforts to develop a pan-Canadian registry will meet this need.

### Chapter 2: Kidney Manifestations

#### Blood Pressure

KDIGO recommends targeting systolic blood pressure (SBP) < 120 mmHg in patients with ADPKD over age 50 using standardized office blood pressure BP measurement (Recommendation 2.1.4), aligning with 2021 KDIGO Clinical Practice Guideline for the management of Blood Pressure in Chronic Kidney Disease^14^ and the 2024 Clinical Practice Guideline for the Evaluation and Management of CKD.^15^ In 2025, Hypertension Canada recommended an SBP target of < 130 mmHg, based on several factors, including feedback from care providers and patients.^16^ People with ADPKD were not included in SPRINT (Systolic Blood Pressure Intervention Trial), due to the results of the HALT-PKD trial.^17^ SPRINT studied people with an age of at least 50 years, hypertension, and at least one other cardiovascular risk factor. SPRINT showed that targeting a mean SBP to < 120 mm Hg (vs. < 140 mm Hg), as assessed by standardized office BP measurement, is associated with a reduction in the incidence of cardiovascular events and all-cause mortality, but with no difference in kidney outcomes. This weaker recommendation (level 2C) is due to low certainty of evidence regarding the optimal BP target in late stages of ADPKD. In SPRINT, targeting a mean SBP < 120 mmHg was associated with an increased risk of adverse events, including hypotension, syncope, electrolyte abnormalities, and AKI, but not injurious falls. The HALT-PKD A trial enrolled only patients with an age below 50 years, and is not relevant to this recommendation.^18^ The HALT-PKD B trial enrolled patients with a mean age of 49 (standard deviation [SD] 8), and a mean eGFR 48 (SD 12) and aimed for a target BP of 110-130/70-80 using an either monotherapy or dual blockade with angiotensin-converting–enzyme (ACE) inhibitor or angiotensin II–receptor blocker (ARB).^19^ Use of monotherapy achieved the target BP in most patients and was well tolerated.

The KDIGO Workgroup placed a high value on reducing the risk of cardiovascular events and all-cause mortality using this SBP <120 mmHg target, while recognizing that targeting a mean SBP of <120 mmHg (vs. 140 mmHg) carries potential risks of harm. Thus, the adaptation of an SBP target of <120 mmHg requires shared decision-making between individual patients and healthcare providers.

Our workgroup supports KDIGO’s statement that healthcare providers should be aware that the target goal is a mean SBP of <120 mm Hg for most people, and flexibility needs to be exercised to accommodate people who cannot achieve this target, due to adverse side effects.

Hypertension Canada also recommends pharmacotherapy initiation for hypertension for adults with BP ≥ 140/90 mmHg and for adults with systolic BP 130– 139 mmHg at high cardiovascular disease risk, which includes diabetes or CKD (strong recommendation, high-certainty evidence).^16^ Therefore, in Canada, pharmacologic treatment should be used in combination with lifestyle modification. A target SBP of < 130 mmHg should be decided and adopted after shared decision making, and subject to periodic review or in response to adverse events.

Our Canadian workgroup supports standardized office BP measurements (Recommendation 2.1.1) and complementing them with out-of-office readings (Recommendation 2.1.2), consistent with KDIGO and Hypertension Canada guidelines for CKD.^14,16^

Hypertension management is particularly important in young high-risk patients with ADPKD, such as those that were enrolled in HALT-PKD A trial where guidelines suggest more aggressive targets (110/75 mmHg) (Recommendation 2.1.3). Providers should ensure standardized measurement techniques are used, and patients should receive clear instructions on home BP monitoring.

Finally, within the Canadian healthcare system, ADPKD patients at an earlier stage of disease may interact more frequently with family physicians, pharmacists, and nurse practitioners than with nephrologists, so these professionals play a critical role in the implementation of this guideline, and targeted knowledge dissemination to these other providers will be essential in ensuring consistent and effective BP management. Our workgroup advocates that policymakers should work to remove potential financial barriers and enable equitable access to home BP monitoring and 24-hour ambulatory blood pressure monitoring.

#### Chronic Pain

**Figure 3.**
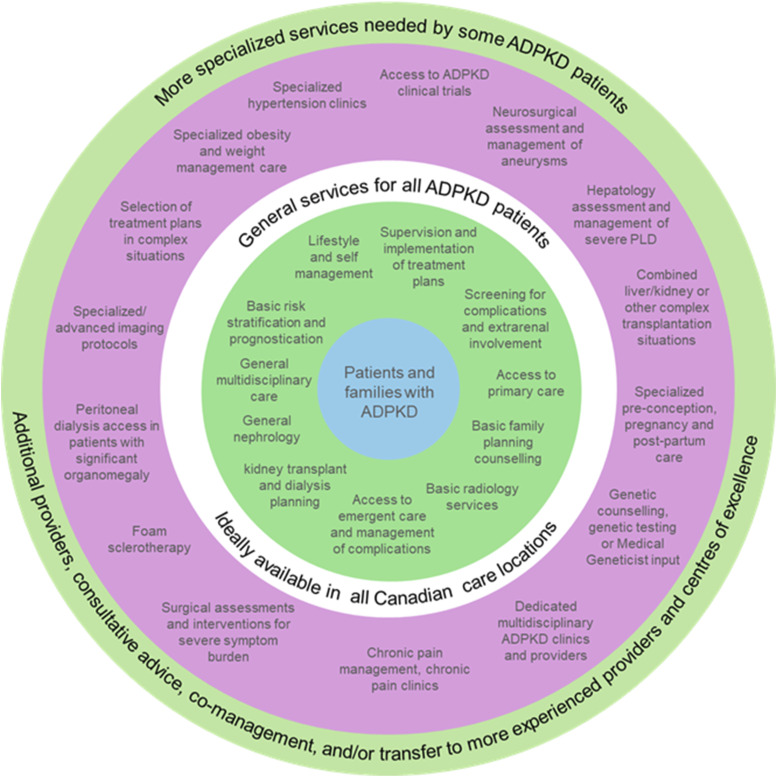
Strata of specialized services in ADPKD care. Inner circle includes services that should be locally available for all Canadians affected by ADPKD, while outer ring includes services that could be provided on a consultation or referral basis

Our Canadian workgroup values KDIGO’s patient-centered pain management guidance (practice points 2.2.1-10) but notes limited access to specialized pain clinics and interventional procedures (such as foam sclerotherapy, renal denervation, and transcatheter arterial embolization), particularly in rural or remote locations. We advocate for policy makers to enable equitable access to these services. In the current state, our workgroup recommends a stepwise approach: start with local options, consult specialists as needed, and refer to tertiary centers with expertise in advanced pain management and intervention options for difficult cases. Our workgroup emphasized shared decision-making and informed patient choice, as some patients may prefer local procedures despite higher recurrence risk such as possible in cyst aspiration without sclerotherapy (Figure 3).

#### Cyst Infections and Kidney Stones

Cyst infections should be treated for 4-6 weeks (Recommendation 2.6.5). Providers and patients should be encouraged to contact their nephrologist to discuss the treatment of cyst infections, especially if the treatment plan was initiated in a non-nephrology setting (e.g. emergency department), which is often the case. Urine and blood cultures should be obtained in combination with imaging. Treatment with a lipid-soluble antibiotic (e.g., fluoroquinolones or trimethoprim-sulfamethoxazole) is recommended (Practice point 2.6.5). In ADPKD, the signs and symptoms of cyst infection and pyelonephritis are often indistinguishable, and thus the longer duration of treatment ensures that a cyst infection will not be undertreated. Occasionally, drainage of an infected cyst may be needed. In these situations, it may be advisable to seek the opinion of an ADPKD centre of excellence that can provide access to imaging and drainage.

While obstructing kidney stones can be complex in ADPKD and are ideally managed by centers with ADPKD-specific expertise, our workgroup recognizes that timely intervention is critical, and not all patients are treated in settings with extensive expertise. We suggest straightforward cases be managed locally when possible but also encourage local practitioners to seek consultative management advice from urologists in specialized centers, including discussion of when to refer to a center of expertise for complex cases that cannot be managed safely or effectively within local resources. Dietary assessments and counselling may be helpful in reducing kidney stones, but there may be some challenges in accessing dietitian support.

### Chapter 3: Kidney Transplantation & Kidney Replacement Therapy

#### Transplantation

Our Canadian workgroup agrees that the weight estimated from total liver and kidney volumes should ideally be subtracted to obtain accurate BMI calculations for consideration of kidney transplantation (Practice point 3.2.7). However, liver volume and weight measurements are not readily available on most radiology reports, an area of potential improvement that would provide valuable information.

The decision to perform a nephrectomy and the timing of the surgery are joint decisions between the patient, nephrologist, and surgeon. These decisions have limited evidence and are often guided by patient symptoms and considerations regarding the timing of dialysis initiation. Our workgroup notes that variation in approaches exists across Canada. Moreover, the approach differs between patients anticipating a living compared to a deceased donor kidney transplant. For recipients of living donors, if bilateral nephrectomies are performed pre-transplant, the patient needs to start kidney replacement therapy. Even a unilateral nephrectomy may precipitate earlier dialysis initiation. In general, pre-transplant nephrectomy is only considered in situations where kidney volume is substantial and physical space would challenge a kidney transplant, or for debilitating space-occupying symptoms. Unilateral compared to bilateral nephrectomies are also dependent on the surgeon and centre. Bilateral nephrectomies can lead to prolonged post-operative hypotension, which can put the transplant graft at risk. Occasionally, a contralateral nephrectomy may be required if unilateral is done initially for space considerations. Recurrent ascites and serositis following bilateral nephrectomies have been reported.^20^

#### Peritoneal Dialysis

Peritoneal dialysis (PD) is feasible in ADPKD (Recommendation 3.3.1), and ADPKD should not be considered a relative or absolute contraindication to PD. A higher risk of abdominal wall hernias exists in ADPKD patients, but there is no difference in abdominal wall hernias between ADPKD patients treated with PD as compared to hemodialysis. Compared to patients on PD with any cause of kidney failure, there is no difference in overall survival, PD technique survival, PD adequacy, or infections.^21^ Moreover, home therapies are the preferred treatment for kidney failure in Canada.

### Chapter 4: Therapies to Delay Progression

#### Tolvaptan

Tolvaptan is the only ADPKD-specific cyst-targeted therapy approved (Recommendation 4.1.1.1). Our Canadian workgroup agrees with KDIGO practice points about individualizing the risk-benefit to tailor treatment recommendations for individual patients, including using MIC. However, KDIGO recommendations are weighted towards MIC and eGFR, while our Canadian workgroup felt individualized risk-benefit discussions are needed, especially for moderate-risk patients (MIC 1C), where genetic testing, eGFR decline, family history, and presence of complications inform decisions as outlined in a previous Canadian consensus guidance.^22^ Older patients (i.e. over 55 years of age) with slower decline in eGFR may yield a smaller benefit-risk ratio, but if avoidance of kidney failure is strongly desired in older patients with progressive disease they may still desire treatment with tolvaptan after an appropriate discussion of the risks and benefits (Practice point 4.1.1.1).

Some Canadian experts report measuring urine osmolarity in tolvaptan-treated patients as urine osmolarity can provide information regarding clinical response and adherence. Nonetheless, we agree that the standard approach should be to up-titrate to the maximum tolerated dose (90 mg upon waking and 30 mg 8 hours later) as was done in the seminal ADPKD trials of tolvaptan (Practice point 4.1.3.3). A post-hoc analysis suggested a more favourable response in those who experienced a greater drop in urine osmolality (to below 280 mOsm/L) but this may only represent a marker of treatment adherence.^23^ Tolerability and aquaretic symptom burden should be the main factors considered when deciding whether to escalate or decrease the dose and routine measurement of urine osmolarity has limited clinical utility.

Funding for Tolvaptan varies across Canada but still largely depends upon private drug insurance coverage. Many, but not all, provinces have prior authorization programs to cover the cost for patients with high risk ADPKD who do not have private drug coverage.

#### Somatostatin Analogues

KDIGO reviewed clinical trials to reduce kidney cystogenesis with long-acting somatostatin analogues (octreotide, lanreotide, pasireotide). Despite a reduction in kidney volume growth, there was inconsistent results regarding slowing eGFR decline, leading to them not being recommended (Recommendation 4.6.1). In Canada, somatostatin analogues are not approved for the treatment of ADPKD, so their use would be off-label or targeted to liver cysts (see Chapter 5 below). Availability and cost coverage of somatostatin analogues may also be a barrier. The prescription of somatostatin analogs may be best done in the context of liver cysts and under the expertise of an experienced hepatologist.

### CKD Management

There is limited data regarding the use of sodium-glucose cotransporter-2 (SGLT2) inhibitors in ADPKD, as trials excluded patients with ADPKD citing safety concerns. There is a possible protective hemodynamic and anti-fibrotic effect of inhibiting SGLT2 in ADPKD but concerns for osmotic diuresis promoting increased vasopressin release and cyst growth have also been suggested. Animal studies have had inconclusive results and are reviewed by KDIGO. A small study suggested SGLT2 inhibition may have a beneficial effect in those already treated with tolvaptan.^24^ Multiple trials of SGLT2 inhibitors are underway in patients with ADPKD. The KDIGO practice point recommending against SGLT2 inhibitor use is regarding progression of ADPKD, but SGLT2 inhibition could be considered in the context of different evidence-based indications (i.e. heart failure or diabetic kidney disease, particularly in those with mild ADPKD). Glucagon-like peptide-1 (GLP-1) receptor agonists and mineralocorticoid receptor antagonists (MRA) are both intriguing CKD therapies, but no studies in patients with ADPKD are yet available.

### Ketogenic Interventions

Preclinical studies have suggested that ketogenic interventions may reduce cyst growth by starving the cells that are dependent on aerobic glycolysis.^16^ Unfortunately, clinical trial data in humans are lacking. Concerns around the safety of ketogenic diets in those with ADPKD exist, including dyslipidemia, increased risk of kidney stone formation, and ketoacidosis in patients with impaired GFR. In Canada, patients do not have easy access to these types of supplements, which are also costly. They can be purchased as nutritional supplements and are not regulated as prescription medications. We agree with the KDIGO practice point 4.8.1 that there is insufficient evidence to recommend ketone body supplementation and advise against its implementation until larger RCTs are conducted.

### Chapter 5: Polycystic Liver Disease

Somatostatin analogs led to a modest but significant reduction in total liver volume growth that may reduce symptoms and improve quality of life for those with abdominal pain, distention, early satiety, or shortness of breath from total liver volume, and may reduce the need for liver transplantation (Recommendation 5.2.3.1). There are concerns of an attenuation of benefit or “escape phenomenon” with somatostatin analog use over 1 year, and long-term efficacy needs to be studied. The KDIGO guideline suggests evaluation of liver growth after 6-12 months and stopping the somatostatin analogue if inhibition of growth is not observed (Practice Point 5.2.3.4). However, workgroup members report the determination of total liver volume is not available nor routinely reported on imaging reports in Canada. We were unclear about the optimal way to obtain liver volume from imaging. If total liver volume is a therapeutic target, education and automated tools to facilitate the measurement or estimation of total liver volume are needed.

Prescribers should be aware of the different somatostatin analogs – lanreotide, octreotide, pasireotide – and their side effect profile: gastrointestinal intolerance, hyperglycemia, gallstones, bradycardia, liver cyst infections; all are listed by Health Canada as approved or marketed (Practice Point 5.2.3.3). The access and cost of long-acting somatostatin analogs may represent a challenge in either private or provincial drug coverage plans. Many nephrologist workgroup members are not familiar with the use of somatostatin analogs and would prefer to refer patients to hepatology. Where hepatology support is available, it is unclear if hepatologists or nephrologists are currently managing the treatment of severe polycystic liver disease in those with ADPKD in Canada. Regardless, nephrologists should be aware of the available treatment options. Ideally, centralized regional centres of expertise should be developed to manage severe polycystic liver disease and build experience for this uncommon condition to ensure standardized and high-quality care (Practice point 5.2.3.1).

### Chapter 6: Intracranial Aneurysms

KDIGO recommends intracranial aneurysm screening in people with ADPKD and prior subarachnoid hemorrhage or a positive family history of intracranial aneurysms, subarachnoid hemorrhage, or unexplained sudden death in those eligible for treatment and with reasonable life expectancy (Recommendation 6.1.2). Family history of aneurysm can be difficult to delineate and may be inaccurate. In general, while screening for an intracranial aneurysm is not completely risk-free, it is low-risk. Anxiety regarding discovered small aneurysms must be balanced with the possible catastrophic outcomes of aneurysm rupture. Our Canadian workgroup feels recommendation 6.1.2 represents the minimum screening that should be offered to patients with ADPKD. Patients with ADPKD without a personal or family history of intracranial bleeding may still benefit from screening. Informed shared decision-making with patients is integral. Our Canadian workgroup feels patients with APDKD who are candidates for intervention if an intracranial aneurysm is found should be offered counseling around their risk of intracranial aneurysm, the proposed screening method, and what may occur if an intracranial aneurysm is found (Practice point 6.1.5). After appropriate counseling, patients should be offered the opportunity to decide on intracranial aneurysm screening for themselves (Practice point 6.1.8).

In the Canadian context, access to MRI is disparate. Some centers have efficient and rapid access, while others have long wait times or limited access. Alternatives to MRI, include CT angiogram, which often provides better vascular imaging but exposes the patient to radiation and may carry risks associated with contrast exposure, especially in patients with lower eGFR. In those patients where no intracranial aneurysm is found, repeat screening is recommended every 5-10 years (Recommendation 6.1.11). KDIGO guidance suggests that aneurysm screening should be based on risk; however, as a validated method of aneurysm risk assessment does not exist, this remains a significant knowledge gap. There is also an opportunity for translational research aimed at identifying non-invasive predictors to differentiate those who may be at risk of developing intracranial aneurysm rupture in the context of ADPKD.

### Chapter 7: Lifestyle and Psychosocial Aspects

Our Canadian workgroup agreed on the importance of self-management education. Further efforts to support and promote patient support, education, and awareness groups are needed (Table 1).

Significant psychosocial burdens including anxiety, chronic pain, and financial stress, are a reality in people with ADPKD. Our workgroup feels individualized discussions regarding physical activity, strength training, and contact sports (eg. hockey) are required (Practice point 7.2.2).

Cannabis has been legalized in Canada since 2018, but our understanding of the pharmacokinetics in advanced kidney disease and its impact on chronic pain in APDKD remains limited. Risk of cannabis contamination (i.e. fentanyl) is real when obtained from unregulated sources (Practice point 7.3.4.1).

### Chapter 8: Pregnancy and Reproductive Issues

Hormone therapy, and estrogen in particular, is associated with liver cyst growth (Practice point 8.1.2). Canadian nephrologists should obtain volumetric liver imaging, ideally with MRI or CT, to inform discussions about contraception, in vitro fertilization, or hormone replacement. Estrogen avoidance would also impact individuals seeking gender affirming hormone therapy. In the Canadian context, access to MRI may be difficult for this purpose, and there are concerns around repeated CT scans. Ultrasound may help inform the initial decision, but it may be insufficient for serial follow-up or nuanced discussions related to liver size.

Our Canadian workgroup feels there is room for improvement in preconception counseling (Practice point 8.2.1), for both men and women. Access to preimplantation genetic testing is variable and often costly in Canada. Virtual consultation may be used to help facilitate interactions. Access to multidisciplinary teams with this expertise may not exist or be limited. The composition of pre-conception counseling teams described by KDIGO is unclear, as are their roles. Additionally, it is not well-established that counselling is the responsibility of nephrologists versus other care providers. Special consideration should be given to offering aneurysm screening before pregnancy in accordance with practice points in Chapter 6 (Practice point 8.2.5). Awareness of opportunities and referral to centers of excellence is recommended for patients seeking additional advice.

Frequent assessment of kidney function and proteinuria is standard care in patients with ADPKD who are pregnant, but access to soluble fms-like tyrosine kinase-1-to-placental growth factor ratio (sFlt-1/PlGF) (Practice Point 8.3.2) is severely restricted in Canada. Even ADPKD centers of excellence have difficulty accessing sFlt-1/PlGF, particularly if sequential monitoring is required. Further, the role of sFlt-1/PlGF testing is not established in patients with ADPKD and normal GFR. In Canada, patients with ADPKD who are pregnant should be followed by multidisciplinary teams with expertise in medical disorders of pregnancy and high-risk obstetrics; in locations where physical access to these services is limited, those specialized centres can provide consultative advice to the local pregnancy care providers. The purpose of these teams is to monitor patients through their pregnancy, provide appropriate pre-eclampsia prophylaxis, and deal with the potential of both ADPKD and non-ADPKD complications that may arise. We agree that proteinuria should be followed with random protein quantification, and dipstick assessment alone is insufficient.

## Chapter 9: Pediatric Issues

Annual 24-hour ambulatory blood pressure testing is recommended after the age of 5 years (Practice point 9.2.2). Our Canadian workgroup feels the KDIGO guidance to avoid routine screening for extrarenal manifestations in pediatric ADPKD cases is too prescriptive (Practice point 9.3.2). However, we do not recommend routine aneurysm screening as rupture is an extremely rare event in children.^25^ Individual cases may dictate additional evaluation based on symptoms, family history, or parental concerns. Significant effort has been focused on the development of robust transitional programs, ideally with a transitional period before the full transfer of care to an adult provider (Practice point 9.5.2), but we recognize they are not uniformly available across Canada.

### Chapter 10: Management of People With ADPKD

Our Canadian workgroup strongly supports calls for broader engagement of people with ADPKD in research initiatives (Practice point 10.3).^26^ We highlighted the increasing contributions of Canadian patient partners to research design and implementation in Canada such as the CanSOLVE-CKD and the Canadian National Trial Network (CNTN).^27^ However, we note an absence of a national ADPKD registry in Canada. A lack of a national registry is a significant barrier to a better understanding of disease prevalence, natural history, and care gaps in the Canadian context. The ideal solution would be a national registry that integrates patient-led enrollment and self-reporting tools with center-led clinical data capture, aligning with international standards and actively engaging underrepresented populations—including Indigenous patients and young adults transitioning from pediatric care.

Continued education of nephrologists caring for individuals with ADPKD is needed, who must have the literacy, resources, and authority to implement genetic testing (Practice point 10.4). Many nephrologists with expertise in ADPKD currently cannot order genetic tests independently due to restrictive local policies, resulting in unnecessary delays in diagnosis. Improved collaboration with colleagues in Medical Genetics or specialized Nephrology genetics clinics may reduce this barrier. We are encouraged by the emergence of NephroGenetics fellowship programs in Canada and advocate for their expansion nationwide and recognition by the Royal College of Physicians and Surgeons of Canada.^28^ The integration of genetics-focused continuing medical education (CME) into major nephrology meetings and societies would further support workforce readiness. A pan-Canadian virtual consultation model (e.g., a kidney genetic e-consult service) would be invaluable to help community nephrologists navigate testing, risk stratification, and counselling.

Our Canadian workgroup recognizes the need for standardized data definitions across Canadian provinces, and these must be aligned with existing international registries. It would be beneficial for patients and physicians to have access to a database or care pathway diagram listing ADPKD centers of excellence (and specific services available) in all major Canadian cities. We provide an initial listing in Supplemental Table 1.

Finally, our Canadian workgroup encourages the use of peer support resources such as those offered by the PKD Foundation of Canada and the Kidney Foundation of Canada to support patient empowerment, education, and self-management.

### Additional Points not Addressed in the KDIGO ADPKD Guidelines

Identifying issues not raised can be more challenging than assessing the provided recommendations and practice points. Increasing the awareness of ADPKD as a condition that is present in individuals of all ancestral groups, including indigenous populations, was felt to be an omission. Raising awareness of the possibility of ADPKD in all populations is needed. Moreover, rural and indigenous populations may have limited access to sub-speciality assessment and care. Many specialist ADPKD providers have access to sub-specialty procedures and additional testing not available to all providers. There are growing opportunities for use of virtual platforms to provide advice and counselling to patients and providers in centers with less access to specialist services.

## Conclusion

**Figure 4.**
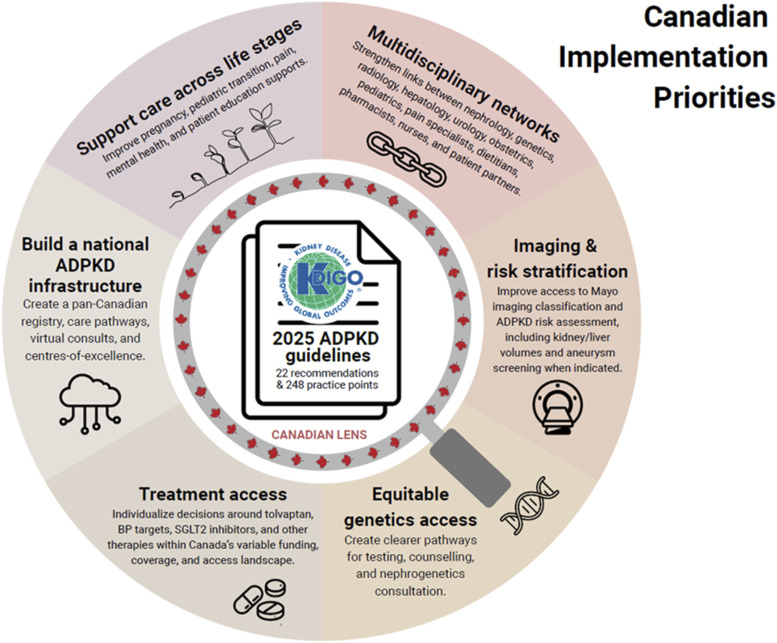
A focused view of KDIGO ADPKD guideline implementation through a Canadian lens

This Canadian workgroup enthusiastically endorses the KDIGO guidelines and highlights key Canadian opportunities (Figure 4): improvements in multidisciplinary care networks, improved access to genetic testing, and refinement of the role of CKD therapies in ADPKD management.

## Supplemental Material

Supplemental Material - Canadian Society of Nephrology Commentary on the 2025 Kidney Disease Improving Global Outcomes Clinical Practice Guidelines for Autosomal Dominant Polycystic Kidney Disease Supplemental Material for Canadian Society of Nephrology Commentary on the 2025 Kidney Disease Improving Global Outcomes Clinical Practice Guidelines for Autosomal Dominant Polycystic Kidney Disease by Matthew B. Lanktree, Nick Ashawasega, Micheli Bevilacqua, Daniel G Bichet, Guillaume Bollée, Pierre-Antoine Brown, Louis Girard, Paul Goodyer, Jay Hingwala, Mathieu Lemaire, Philip McFarlane, Louise Moist, York Pei, Normand Proulx, Clara Schott, Steven Soroka, Caitlyn Vlasschaert, Nicholas Watkins, and Ahsan Alam in Canadian Journal of Kidney Health and Disease.

## References

[1] Kidney Disease: Improving Global Outcomes (KDIGO) ADPKD Work Group . KDIGO 2025 clinical practice guideline for the evaluation, management, and treatment of autosomal dominant polycystic kidney disease (ADPKD). Kidney Int. 2025;107(2S):S1-S239. 39848759 10.1016/j.kint.2024.07.009

[2] Zahran S Mathew A Harrison TG Jauhal A Hladunewich MA Mustafa RA . The Canadian Society of nephrology methods for developing and adapting clinical practice guidelines: An update. Can J Kidney Health Dis. 2025;12:20543581251346070. 10.1177/20543581251346074 PMC12214302 40606775

[3] ACMG Laboratory Quality Assurance Committee Richards S Aziz N Bale S , et al. Standards and guidelines for the interpretation of sequence variants: a joint consensus recommendation of the American College of Medical Genetics and Genomics and the Association for Molecular Pathology. Genet Med. 2015;17(5):405-424. 25741868 10.1038/gim.2015.30 PMC4544753

[4] Ghanem A Borghol AH Munairdjy Debeh FG , et al. Biomarkers of kidney disease progression in ADPKD. Kidney Int Rep. 2024;9(10):2860-2882. 39435347 10.1016/j.ekir.2024.07.012 PMC11492289

[5] Iliuta I-A Win AZ Lanktree MB , et al. Atypical Polycystic Kidney Disease as defined by Imaging. Sci Rep. 2023;13(1):2952. 36807559 10.1038/s41598-022-24104-w PMC9941465

[6] Devuyst O Pei Y . Next-generation sequencing for detection of somatic mosaicism in autosomal dominant polycystic kidney disease. Kidney Int. 2020;97(2):261-263. 31980075 10.1016/j.kint.2019.11.019

[7] Lanktree MB Kline T Pei Y . Assessing the risk of progression to kidney failure in patients with autosomal dominant polycystic kidney disease. Adv Kidney Dis Health. 2023;30(5):407-416. 38097331 10.1053/j.akdh.2023.06.002

[8] Cornec-Le Gall E Alam A Perrone RD . Autosomal dominant polycystic kidney disease. Lancet. 2019;393(10174):919-935. 30819518 10.1016/S0140-6736(18)32782-X

[9] Borghol AH Bou Antoun MT Hanna C Salih M Rahbari-Oskoui FF Chebib FT . Autosomal dominant polycystic kidney disease: an overview of recent genetic and clinical advances. Ren Fail. 2025;47(1):2492374. 40268755 10.1080/0886022X.2025.2492374 PMC12020221

[10] Cowan JS Kagedan BL Graham GE Heim-Myers B Bombard Y . Health care implications of the Genetic Non-Discrimination Act: Protection for Canadians’ genetic information. Can Fam Physician. 2022;68(9):643-646. 36100377 10.46747/cfp.6809643 PMC9470184

[11] Bevilacqua MU Hague CJ Romann A , et al. CT of kidney volume in autosomal dominant polycystic kidney disease: Accuracy, reproducibility, and radiation dose. Radiology. 2019;291(3):660-667. 30964424 10.1148/radiol.2019181830

[12] Incident End-Stage Kidney Disease Patients: CORR Data. CIHI [Internet]. [cited 2018 Feb 26]. Available from. https://tinyurl.com/y2xbsvfa

[13] Lanktree MB Haghighi A Guiard E , et al. Prevalence Estimates of Polycystic Kidney and Liver Disease by Population Sequencing. J Am Soc Nephrol. 2018;29(10):2593-2600. 30135240 10.1681/ASN.2018050493 PMC6171271

[14] Kidney Disease: Improving Global Outcomes (KDIGO) Blood Pressure Work Group . KDIGO 2021 clinical practice guideline for the management of blood pressure in chronic kidney disease. Kidney Int. 2021;99(3S):S1-S87. 33637192 10.1016/j.kint.2020.11.003

[15] Kidney Disease: Improving Global Outcomes (KDIGO) CKD Work Group . KDIGO 2024 clinical practice guideline for the evaluation and management of chronic kidney disease. Kidney Int. 2024;105(4S):S117-S314. 38490803 10.1016/j.kint.2023.10.018

[16] Goupil R Tsuyuki RT Santesso N , et al. Hypertension Canada guideline for the diagnosis and treatment of hypertension in adults in primary care. CMAJ. 2025;197(20):E549-E564. 40419299 10.1503/cmaj.241770 PMC12150426

[17] SPRINT Research Group Wright JTJ Williamson JD Whelton PK , et al. A Randomized Trial of Intensive versus Standard Blood-Pressure Control. N Engl J Med. 2015;373(22):2103-2116. 26551272 10.1056/NEJMoa1511939 PMC4689591

[18] HALT-PKD Trial Investigators Schrier RW Abebe KZ Perrone RD , et al. Blood pressure in early autosomal dominant polycystic kidney disease. N Engl J Med. 2014;371(24):2255-2266. 25399733 10.1056/NEJMoa1402685 PMC4343258

[19] HALT-PKD Trial Investigators Torres VE Abebe KZ Chapman AB , et al. Angiotensin blockade in late autosomal dominant polycystic kidney disease. N Engl J Med. 2014;371(24):2267-2276. 25399731 10.1056/NEJMoa1402686 PMC4284824

[20] Umles NS Topf JM Henderson HL Bellovich KA . Idiopathic recurrent serositis after bilateral nephrectomy in ADPKD: PUB219. J Am Soc Nephrol. 2023;34(11S):1109.

[21] Munairdjy DFG Ghanem A Rangarajan V , et al. Multicenter insights into peritoneal dialysis for ADPKD: Role of cumulative cystic organ volumes in treatment complications. Kidney360 [Internet]. 2025. [cited 2025 Sept 17]; Available from. doi:10.34067/KID.0000000888. PMC12626652 40622773

[22] Soroka S Alam A Bevilacqua M , et al. Updated Canadian Expert Consensus on Assessing Risk of Disease Progression and Pharmacological Management of Autosomal Dominant Polycystic Kidney Disease. Can J Kidney Health Dis. 2018;5:2054358118801589. 30345064 10.1177/2054358118801589 PMC6187423

[23] Devuyst O Chapman AB Gansevoort RT , et al. Urine osmolality, response to tolvaptan, and outcome in Autosomal Dominant Polycystic Kidney Disease: Results from the TEMPO 3:4 trial. J Am Soc Nephrol. 2017;28(5):1592-1602. 27920153 10.1681/ASN.2016040448 PMC5407721

[24] Minatoguchi S Hayashi H Umeda R Koide S Hasegawa M Tsuboi N . Additional renoprotective effect of the SGLT2 inhibitor dapagliflozin in a patient with ADPKD receiving tolvaptan treatment. CEN Case Rep. 2024;13(5):419-424. 38494546 10.1007/s13730-024-00859-1 PMC11444039

[25] Walker EYX Marlais M . Should we screen for intracranial aneurysms in children with autosomal dominant polycystic kidney disease? Pediatr Nephrol. 2023;38(1):77-85. 35106642 10.1007/s00467-022-05432-5 PMC8807382

[26] Elliott MJ McCarron TL Schick-Makaroff K Getchell L Manns B Fernandez N . The dynamic nature of patient engagement within a Canadian patient-oriented kidney health research network: Perspectives of researchers and patient partners. Health Expect. 2023;26(2):905-918. 36704935 10.1111/hex.13716 PMC10010076

[27] Murdoch A Tennankore KK Bohm C , et al. Re-envisioning the Canadian Nephrology Trials Network: A can-SOLVE-CKD stakeholder meeting of patient partners and researchers. Can J Kidney Health Dis. 2021;8:20543581211030396. 34345433 10.1177/20543581211030396 PMC8283045

[28] Lanktree MB Shurrab S Ejaz R , et al. Educating the next-generation expert in nephrology genetics. Semin Nephrol. 2025 Sept. 2025;45(5):151648. 10.1016/j.semnephrol.2025.151648 40670224

